# Electrogastrography measurement systems and analysis methods used in clinical practice and research: comprehensive review

**DOI:** 10.3389/fmed.2024.1369753

**Published:** 2024-07-01

**Authors:** David Oczka, Martin Augustynek, Marek Penhaker, Jan Kubicek

**Affiliations:** Department of Cybernetics and Biomedical Engineering, VSB–Technical University of Ostrava, Ostrava, Czechia

**Keywords:** electrogastrography, non-invasive method, measurement systems, electrode placement, measurement apparatus, signal processing

## Abstract

Electrogastrography (EGG) is a non-invasive method with high diagnostic potential for the prevention of gastroenterological pathologies in clinical practice. In this study, a review of the measurement systems, procedures, and methods of analysis used in electrogastrography is presented. A critical review of historical and current literature is conducted, focusing on electrode placement, measurement apparatus, measurement procedures, and time-frequency domain methods of filtration and analysis of the non-invasively measured electrical activity of the stomach. As a result, 129 relevant articles with primary aim on experimental diet were reviewed in this study. Scopus, PubMed, and Web of Science databases were used to search for articles in English language, according to the specific query and using the PRISMA method. The research topic of electrogastrography has been continuously growing in popularity since the first measurement by professor Alvarez 100 years ago, and there are many researchers and companies interested in EGG nowadays. Measurement apparatus and procedures are still being developed in both commercial and research settings. There are plenty variable electrode layouts, ranging from minimal numbers of electrodes for ambulatory measurements to very high numbers of electrodes for spatial measurements. Most authors used in their research anatomically approximated layout with two++ active electrodes in bipolar connection and commercial electrogastrograph with sampling rate of 2 or 4 Hz. Test subjects were usually healthy adults and diet was controlled. However, evaluation methods are being developed at a slower pace, and usually the signals are classified only based on dominant frequency. The main review contributions include the overview of spectrum of measurement systems and procedures for electrogastrography developed by many authors, but a firm medical standard has not yet been defined. Therefore, it is not possible to use this method in clinical practice for objective diagnosis.

**Systematic Review Registration:**

https://www.prisma-statement.org/.

## 1 Introduction

Electrogastrography (EGG) is a diagnostic method used to measure the electrical potentials of the stomach muscles. The method is non-invasive and is measured transcutaneously using electrodes placed on the abdomen over the stomach location (1, 2). The first measurement of the electrogastrogram took place in the early 1920s on animal and human subjects by Professor Alvarez and his team (1, 3). Since then, due to the evolution of measuring instruments and its increasing measurement precision, the research popularity of the method has grown, and many researchers are still interested in this topic (4–9). Although this non-invasive method could complement or even replace current, more invasive diagnostic methods such as gastroscopy or X-ray examination in clinical practice, a medical standard for its consistent measurement has not yet been defined. However, several attempts to achieve its definition have been made by many authors (10–16).

The aim of this study is to analyze the research articles from the first measurements to the present and help future researchers to choose the appropriate equipment and methods to obtain a relevant electrogastrogram. The devices used range from universal bioamplifiers to single-purpose electrogastrographs with very low or very high sampling frequency and analog or digital filtering, which can significantly change the resulting measured signal and its analysis. The overall quality and the possible presence of noise in the signal lead to analysis using methods such as Wavelet transform in the time domain or Fourier transform in the frequency domain but also machine learning methods.

## 2 Review strategy

In this study, a review specifically aimed on existing electrogastrography measurement systems, procedures, and analysis methods in both research and clinical practice is presented. The technique that employed to obtain sources and conduct this review is based on the PRISMA review strategy. Various steps were taken in applying this strategy, including selecting appropriate databases, choosing suitable search terms, applying search criteria, and evaluating the results found. Sources utilized for the creation of this review consisted solely of full papers, original journal papers, conference proceedings, book chapters, other review papers, and graduation theses.

**Figure 1:**
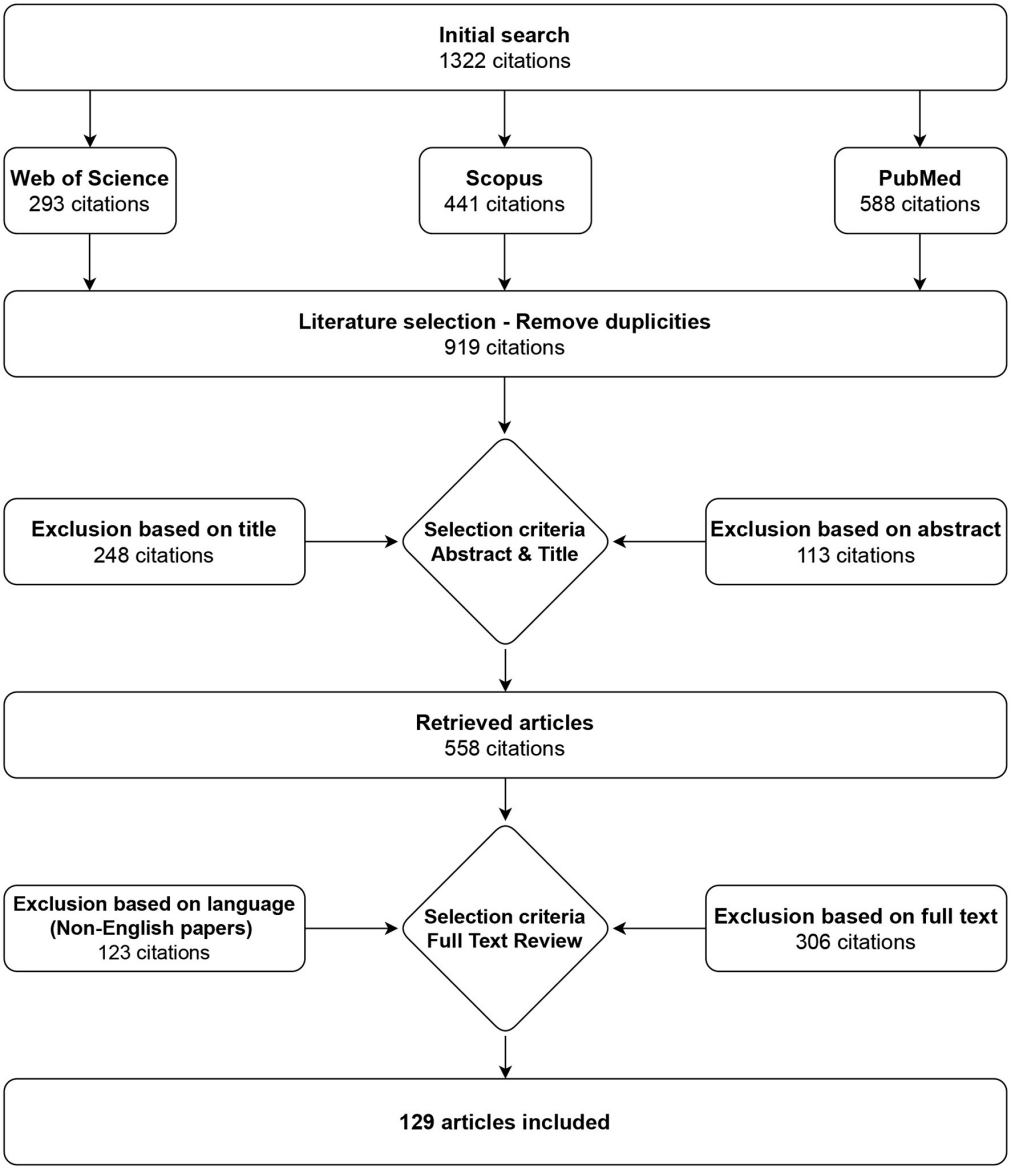
Selection strategy. A total of 129 articles on electrogastrography measurement devices, procedures, filtration, and analysis were identified.

Only papers written in the English language were included, and the Scopus, PubMed, and Web of Science databases were used to search the literature. Query used for search in all mentioned databases was “*gastric myoelectrical OR electrogastrographical OR electrogastrographic OR electrogastrography AND system OR device OR apparatus”*. The results from the three databases were combined, and duplicates and non-English texts were discarded, resulting in 129 matches in July 2023. The selection process is shown in Figure 1.

## 3 Article structure

This study is structured to cover all related areas of assessment of electrical activity of the stomach. First, the principles of electrical activity of the stomach are described in detail, followed by an explanation of measurement and analysis methodologies. Finally, the results from these chapters are discussed and summarized. The overall structure of chapters is shown in Table 1.

**Table 1:** Diagram of the article structure with all sections and subsections.

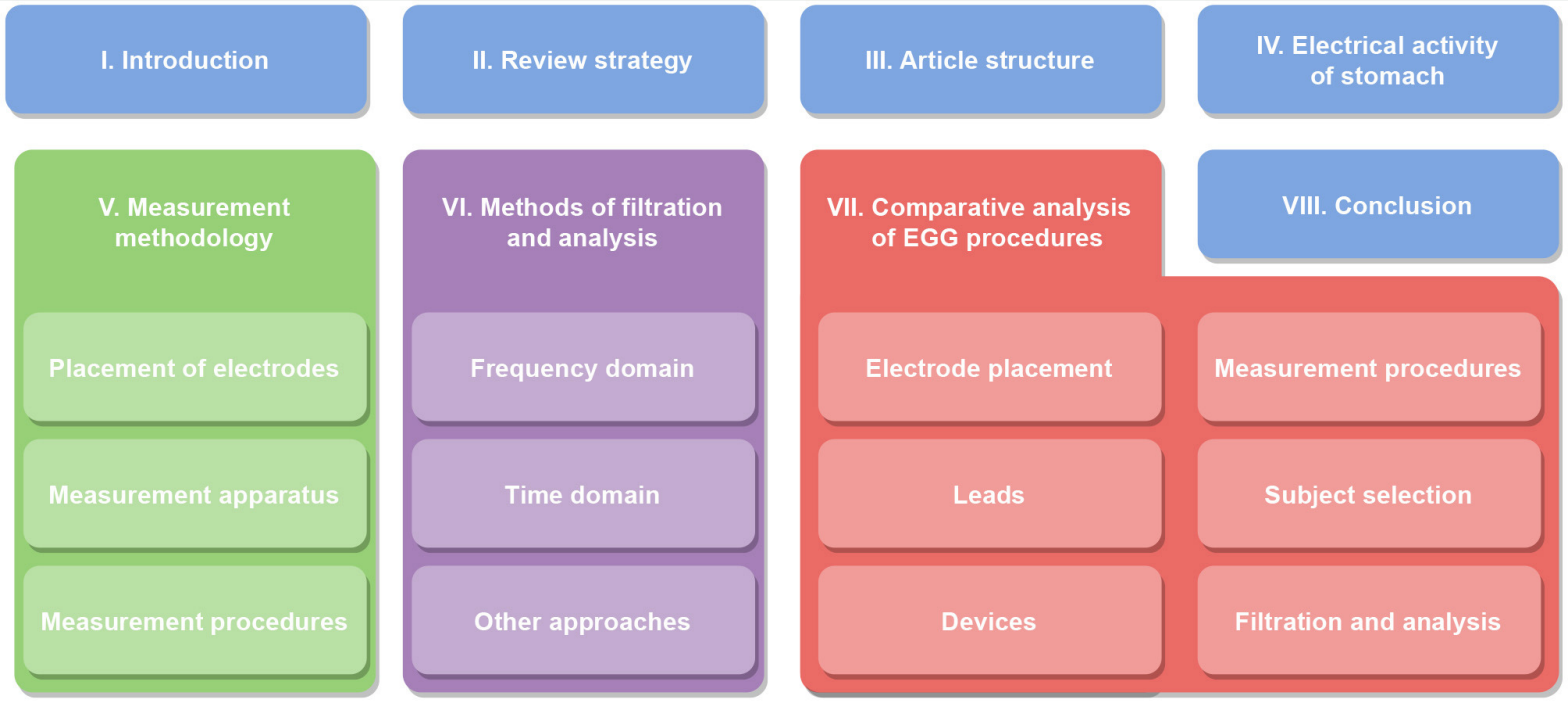

## 4 Electrical activity of the stomach

**Figure 2:**
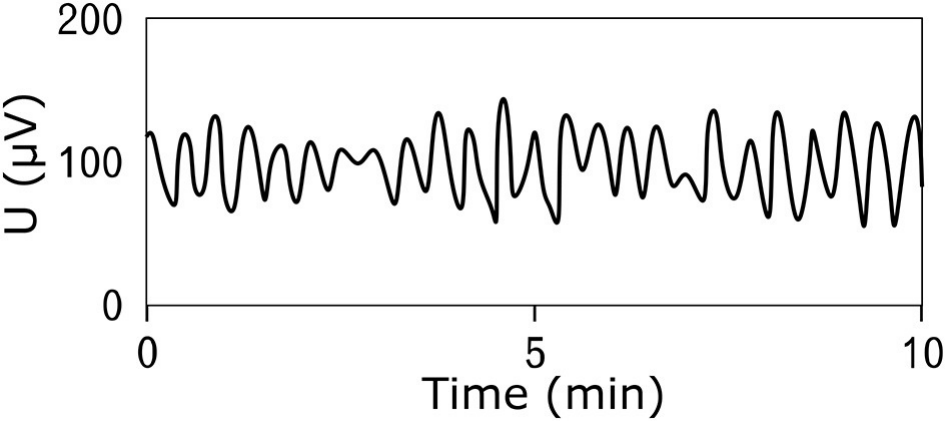
An example of the resting (prepreandial) electrical activity of the stomach (electrogastrogram) of a 22-year-old man obtained by the bipolar method of measurement using an universal bioamplifier. The signal was then low-pass filtered.

**Figure 3:**
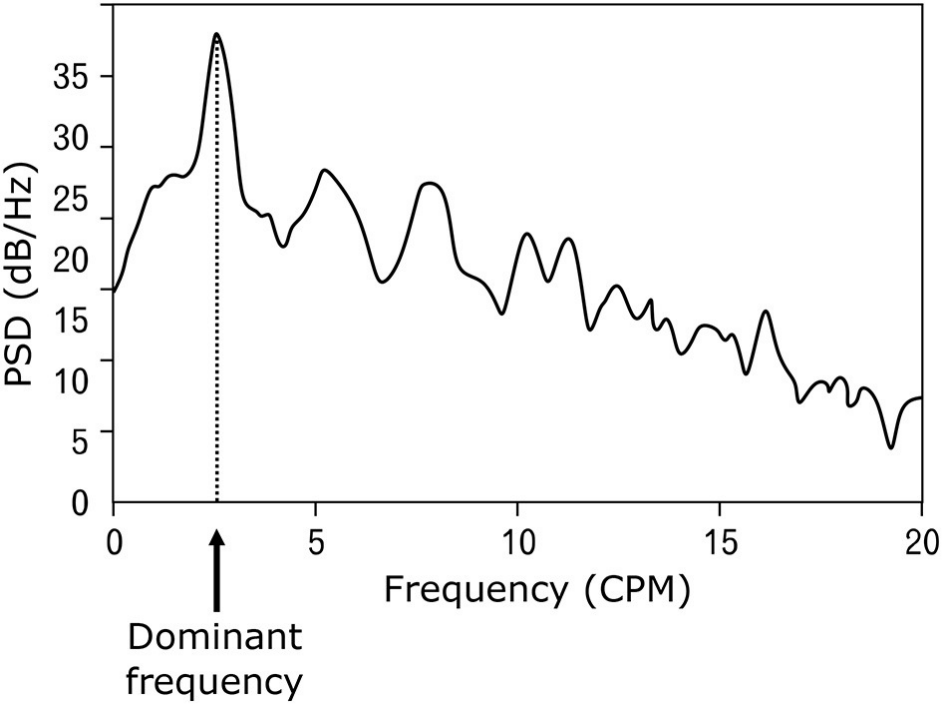
An example of the power spectral density of electrical activity of the stomach.

Electrical potentials that occur on the surface of the stomach are the cause of spontaneous contractions leading to rhythmic activity of the smooth muscles of the stomach (17–19). The frequency of the generated electrical waves is very low, specifically in millihertz; therefore, the common unit describing the signal frequency is cycles per minute (CPM) (2, 20–22). The peak-to-peak voltage of the potentials measured invasively directly on the stomach surface is in the range of 1–2 mV (23–25), and the voltage of signals measured non-invasively on the abdominal wall is in the range of 200–400 μV (24, 26, 27). An example of a normal resting signal of the electrical activity of the stomach is shown in Figure 2. The physiological frequency of gastric electrical waves in the case of an empty stomach is in the range of 2–4 CPM, and in most cases, it is 3 CPM (1, 3, 23–34) as visible in power spectral density spectrum in Figure 3. The electrical activity of an empty stomach is called preprandial, and activity after a meal is called postprandial. The gastric electrical activity originates in places called gastric pacemakers located throughout the body and antrum with different structural characteristics, from which it spreads over the surface of the stomach with different intrinsic frequencies (35). The dominant gastric pacemaker is located in a part of the stomach called the corpus and in its subsection called the greater curvature (31, 36, 37). The electrical activity of the stomach generated by pacemakers originates in the interstitial Cajal cells, as confirmed by the fact that if the cells are removed, the electrical potentials disappear (35, 38–40).

According to several studies, the characteristics of stomach electrical activity change drastically in case of pathological events, such as gastric or duodenal ulcers or stomach carcinoma. While some authors describe changes in the amplitude of the signal, mostly substantial changes occur in the frequency of the signal in preprandial and postprandial states (25, 28, 41, 42). The results from a recent study also suggest that signal frequency can be affected by taste, especially salty and sour tastes (43).

## 5 Measurement methodology

The methodology for measuring electrogastrogram has several steps, such as electrode placement, selecting the measurement instrument, and defining a standardized procedure. To place electrodes, the correct location of the stomach must be identified, the number of electrodes must be chosen, and a specific layout of electrodes must be defined. The measuring apparatus must have standardized parameters to meet the requirements for the acquisition of accurate measurements. The examination procedure must be defined to ensure objectivity and repeatability.

### 5.1 Placement of electrodes

Before the start of measurement, the electrodes must be placed on the abdominal surface to the location supposing overlay of the specific parts of human stomach. However, because the stomach is not visible through the skin, there are several approaches to specify its location reliably or approximately.

#### 5.1.1 Anatomical approximation

**Figure 4:**
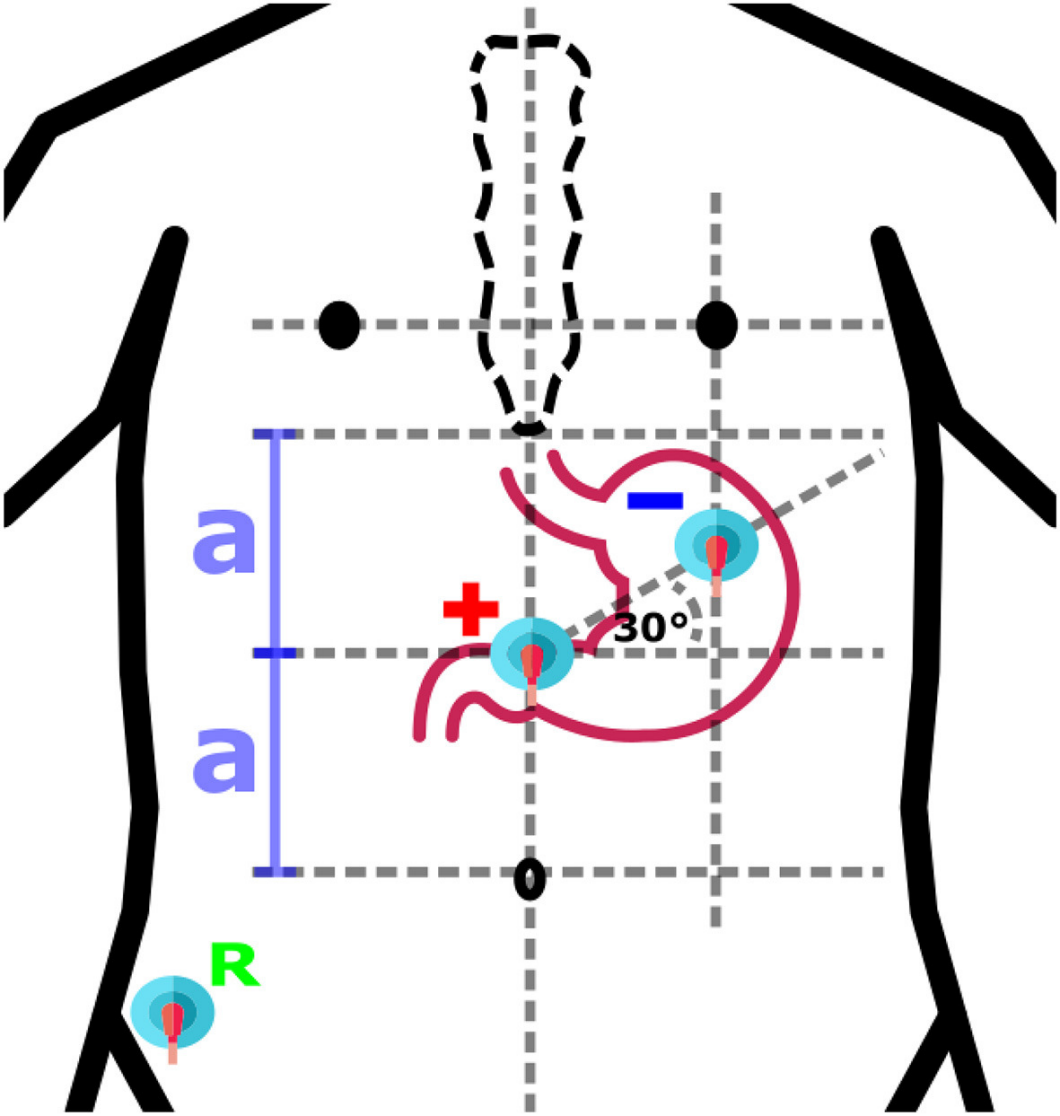
An example of anatomical approximation layout. A bipolar layout consisted of Positive (+), Negative (-), and Reference (R) electrodes is shown. The positive electrode is in the middle of the distance (2a) between the umbilicus and the sternum, while the negative electrode is located at the intersection of the vertical below the left nipple and the 30° line initiated from the location of the positive electrode.

Based on an anatomical similarity of each human being, approximative placement of electrodes could be considered. Locations of electrodes are defined in respect to geometrical relations between anatomical features of human body, while proportional differences between specific individuals are taken into account (4, 12, 26, 44–48). The same approach is used also for other standard diagnostic methods, for example, electrocardiography or electroencephalography. The approach could be considered as the fastest and with potential appropriate use in clinical practice as it does not have any other technical requirements and can be done without attendance of physician. An example of anatomical approximation layout is shown in Figure 4.

#### 5.1.2 Radiography

**Figure 5:**
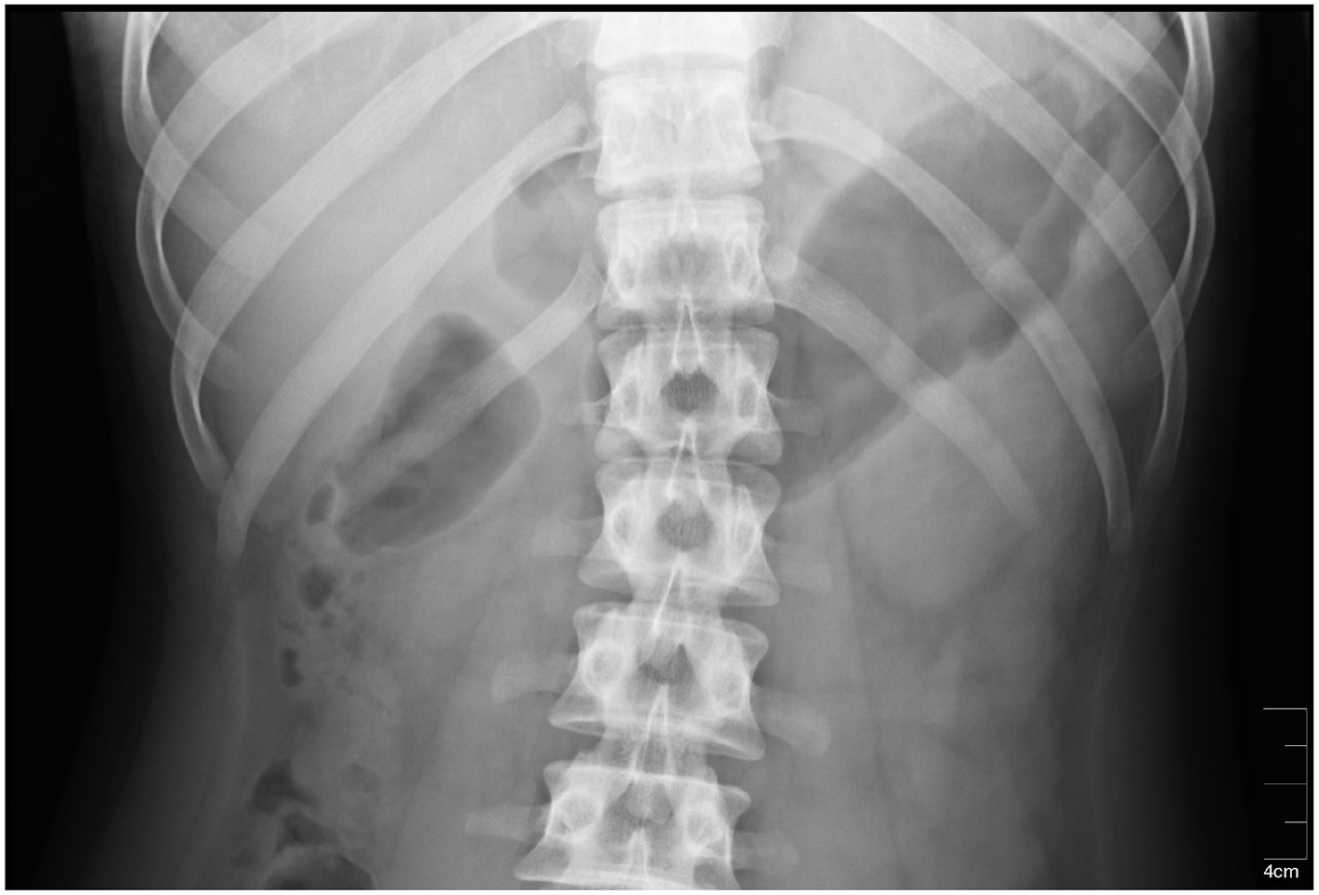
An X-ray image of the stomach. The stomach is located in the dark area on the right side of the radiograph (51).

The stomach location could be also identified using radiography methods which may consist of ingestion or injection of contrast substance such as iodine or barium meal. An x-ray image showing exact position of the stomach in the abdominal cavity is the result of afterward X-ray examination (49, 50). The method is very precise and provides the location of the stomach of an individual subject, especially if taken shortly before actual measurement. However, the subject must undergo another examination, and moreover, he or she must be in contact with ionizing radiation, therefore it is not very suitable for use in clinical practice. An example of an X-ray image is shown in Figure 5.

#### 5.1.3 Ultrasonography

**Figure 6:**
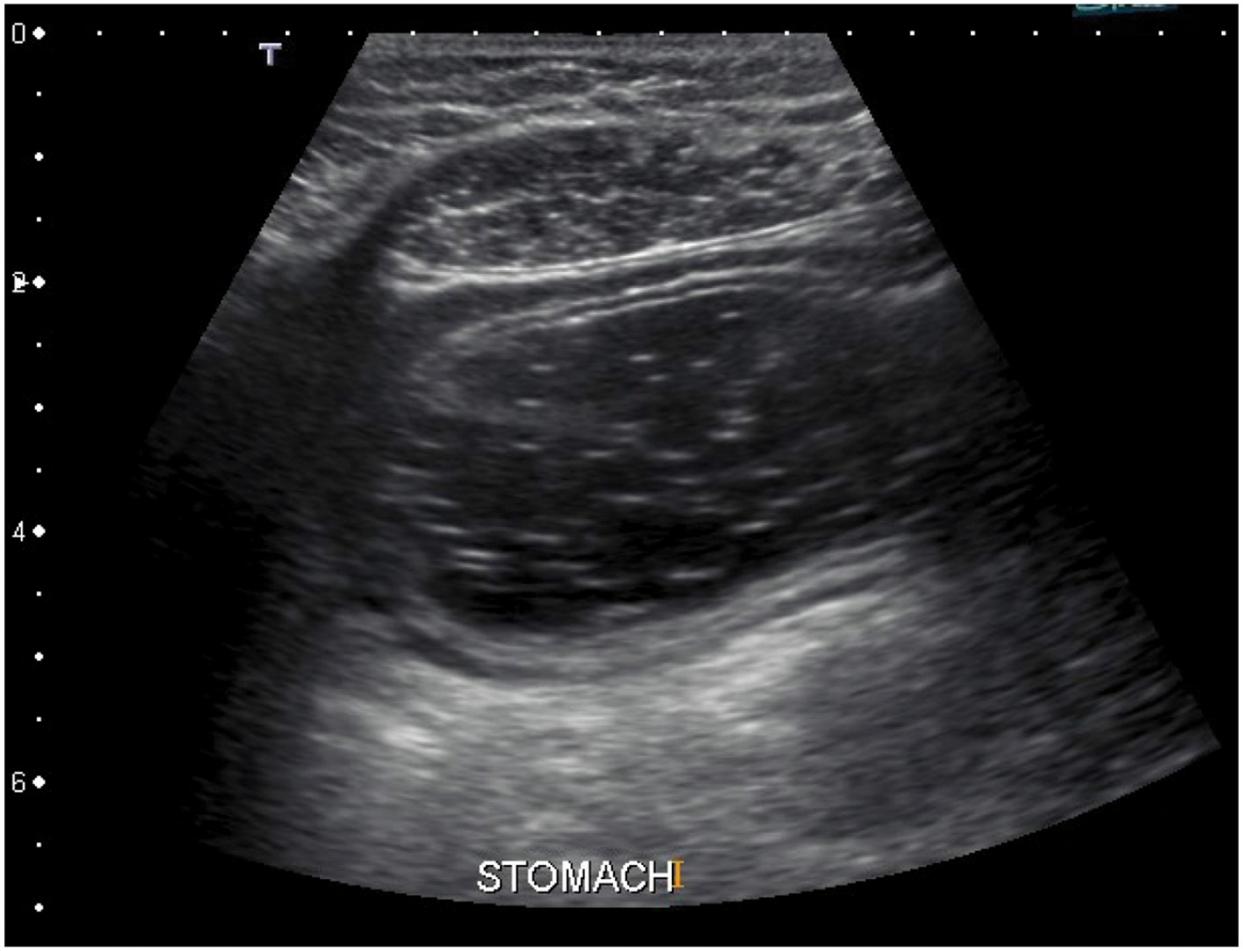
An ultrasound image of the stomach. The stomach is visible as dark area in the middle of the image. The image is taken from transversal plane (57).

Other possible approach could be usage of ultrasound. Ultrasonography is a fast and non-invasive method which allows specification of precise stomach location right on the spot just before the measurement is done (52–56). In comparison with the radiography, the ultrasonography is much safer and more comfortable for examined subjects. The example of an ultrasound image of the stomach is shown in Figure 6, where the stomach is visible as dark area in the middle of the image which is taken from transversal plane. The only problem is that a doctor or at least a well-trained technician is necessary to perform and evaluate such an exam, what drastically increases costs in human resources.

#### 5.1.4 Other imaging modalities

There are also alternative methods such as CT or MRI, which can accurately locate the stomach. However, in comparison to the previously described methods, they demand significantly more time, effort, and financial resources, making them economically inefficient (59–61).

### 5.2 Measurement apparatus

Measurement apparatus consists of electrodes that ensure direct connection better to the skin and measurement device or card performing sampling and leads providing connection between the electrodes and the device.

#### 5.2.1 Electrodes

Historically, zinc electrodes coated with zinc sulfate (Zn/ZnS) were used for measurement of electrical activity of the stomach (1, 3). Technological advancements and inventions of new materials resulted in discovery of silver electrodes coated with silver chloride (Ag/AgCl), and nowadays, this type of electrodes is considered as primary electrodes for the measurement of bioelectric signals. The Ag/AgCl electrodes are also used for the measurement of electrical activity of the stomach (11, 13, 47, 62–64). In most cases, the adhesive floating Ag/AgCl electrodes prefilled with conductive gel are used as they are cheap and easy to use.

Before the electrodes are attached, to increase reliability of electrode contact and reduction of skin-electrode junction impedance, the skin in location of electrode placement should be shaved to remove all hair and gently abraded by sandpaper, and in order to remove grease, it should be cleaned by alcohol at the end (65–68). Measurement should not start immediately after the attachment of electrodes because of the required adaptation of electrode to be fully polarized, which could cause an artifact in other cases (26).

#### 5.2.2 Leads

Electrical connection of leads determines the quality and type of measured signals. Signals could be acquired in unipolar (monopolar) way when electrical potentials are measured between active electrode and reference electrode (47, 64, 69–71). Another way is bipolar acquirement when electrical potentials are measured between two active electrodes with or without use of reference electrode (52, 72–75). Example of one channel bipolar electrode layout with reference electrode is shown in Figure 4. The number of leads used for measurement varies from 1 to an unspecified high number of leads required for multichannel or matrix measurement.

#### 5.2.3 Devices

**Figure 7:**
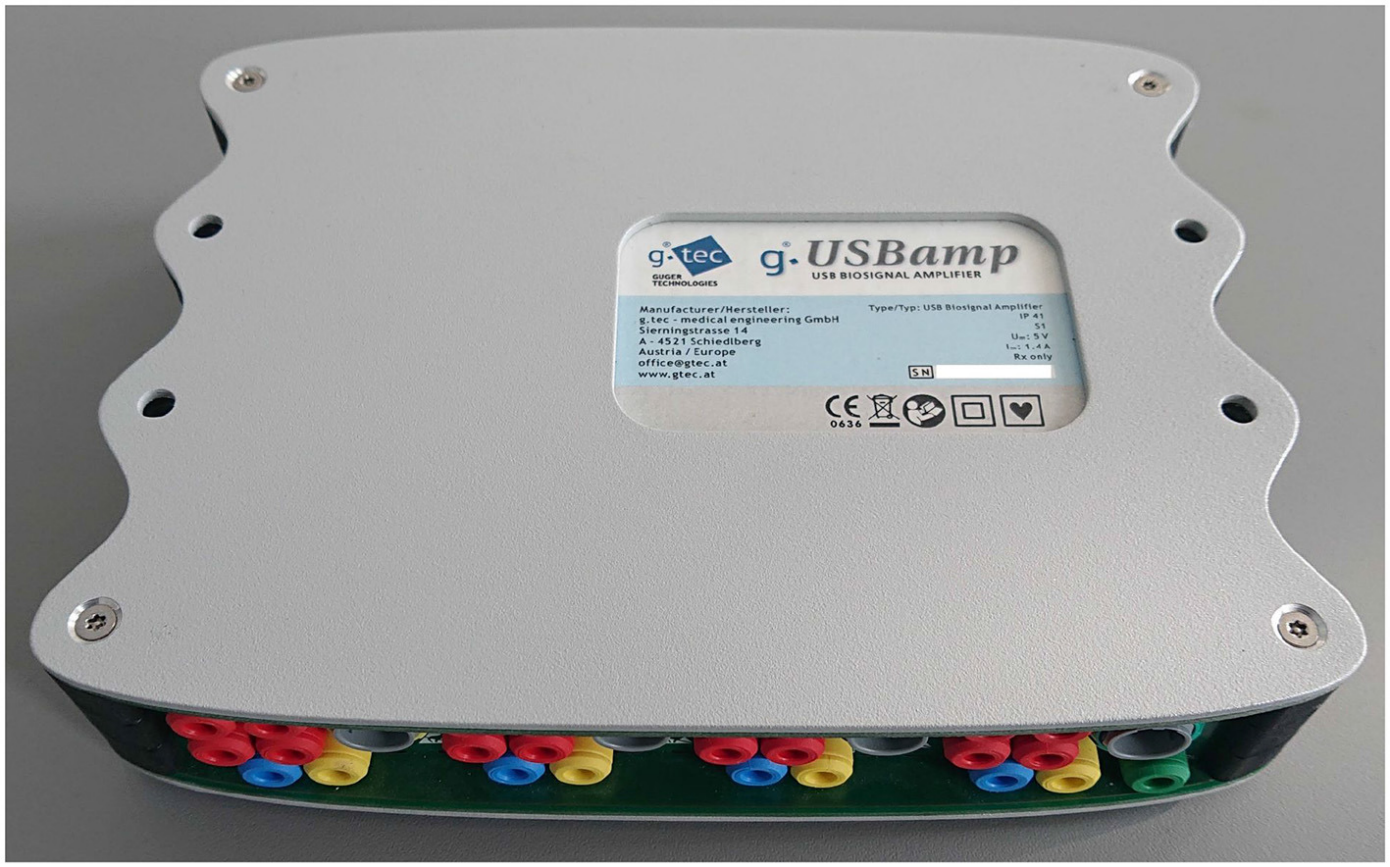
An example of universal bioamplifier with μV precision suitable for electrogastrography measurements (g.USBamp from g.tec) (58).

**Figure 8:**
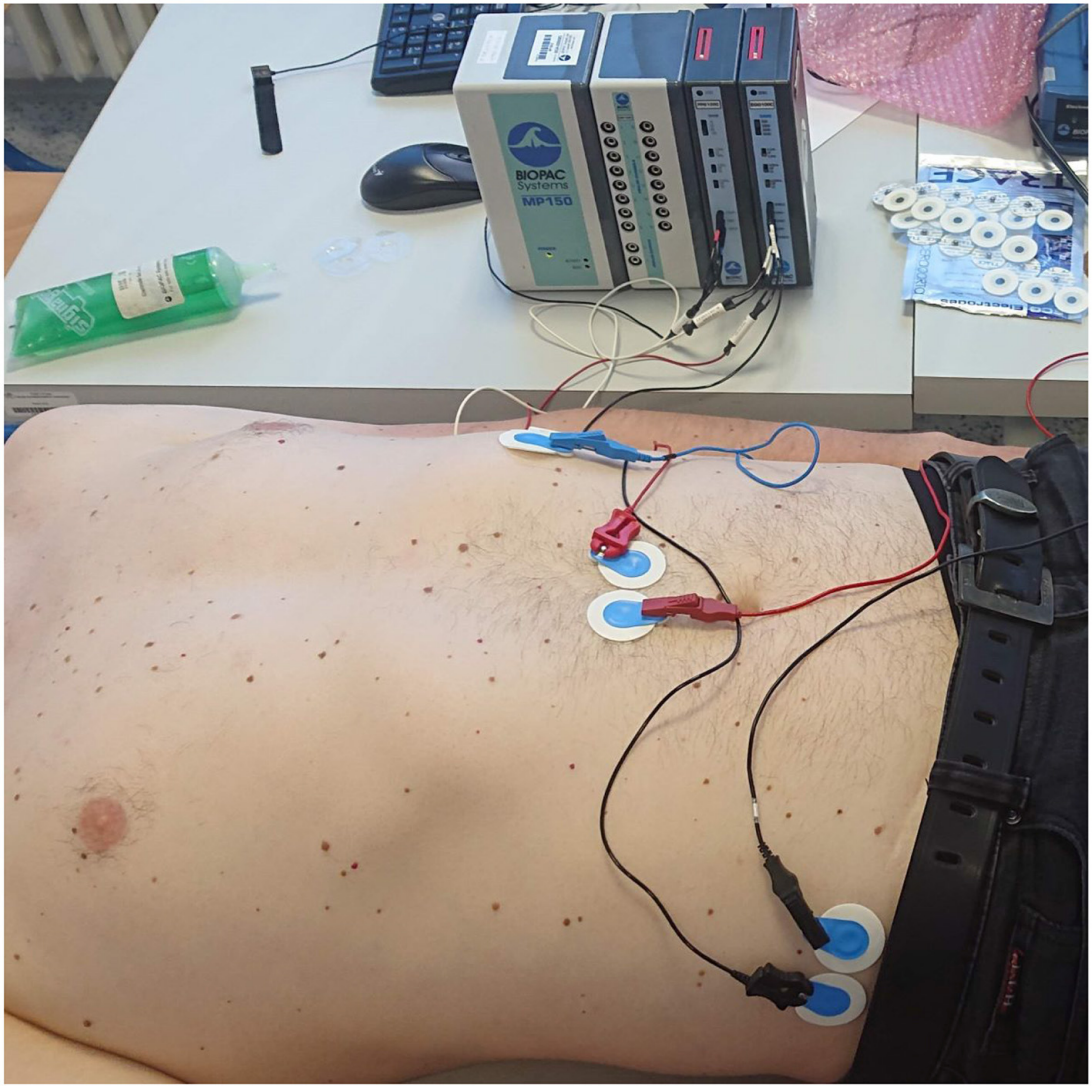
An example of a measuring apparatus consisting of a Biopac electrogastrography module, leads, and disposable electrodes in a comparative experiment with parallel measurement of an electrogastrograph and a universal bioamplifier (the bioamplifier is out of view).

Because of missing standard for measurement, neither of the standard measuring devices is available. There are several commercial systems (Figure 7) or amplifying modules for the acquirement of electrical activity of the stomach. Such systems are from companies such as Synectics Medical, 3CPM, Biopac (Figure 8), Plux, Alimetry, and others. Many authors used commercial systems to acquire gastric signals for their research. For measurement, devices that especially constructed to acquire electrogastrogram (46, 76–79), multipurpose polygraphs (54, 80), or general bioamplifiers (34, 81, 82) are used. Many other authors were also interested in the design and creation of custom systems for measurement of electrogastrogram (10, 11, 13, 83–89). A very important parameter of measurement device is its sampling rate. Sampling rate value may vary from 1 Hz as very sufficient sampling rate regarding the frequency characteristics of signal up to 500 Hz used for high-resolution measurements of stomach electrical activity. However, a higher sampling rate leads to the acquisition of a larger amount of noise component and therefore requires more complex pre-processing (71, 72, 74, 90).

#### 5.2.4 Signal quality

The quality of EGG signals is paramount for accurate interpretation and subsequent clinical or research conclusions. Various studies have employed different methodologies to evaluate and compare the quality of EGG signals. A critical initial step in EGG studies is the acquisition and preprocessing of signals. The quality of EGG signals is heavily dependent on these processes. Authors often compare signal quality based on electrode placement, signal amplification, and filtering techniques. For instance, some studies emphasize the importance of standardized electrode placement on the abdomen to minimize noise and maximize signal integrity. Other researchers focus on the effectiveness of various filtering techniques, such as band-pass filters, to reduce artifacts. The presence of artifacts, such as motion artifacts and respiratory influences, can significantly degrade EGG signal quality. Comparative studies often assess the effectiveness of different noise reduction methods. Studies have mostly evaluated the efficacy of advanced signal processing techniques, such as independent component analysis (ICA) and wavelet transform, in enhancing signal quality (46, 47, 64, 77, 83, 90–93).

Quantitative analysis of EGG signals involves calculating various parameters, such as dominant frequency, power, and the percentage of normal slow waves. Authors compare these parameters to assess the quality and reliability of the EGG recordings. Ultimately, the quality of EGG signals is evaluated based on their clinical relevance and diagnostic utility. Studies often compare the sensitivity and specificity of EGG parameters in diagnosing gastrointestinal disorders. The comparison of EGG signal quality in scientific studies involves a multifaceted approach, considering signal acquisition, preprocessing, noise reduction, quantification, and clinical relevance. Despite the variability in methodologies, a growing consensus on best practices is emerging, which is driven by comparative analyses and reviews. Ensuring high-quality EGG signals is essential for accurate diagnosis and research in gastrointestinal motility disorders. The quality of the acquired signals can be also represented by the signal-to-noise ratio (SNR), which compares the level of the useful signal with its noise. However, this parameter is strongly dependent on the measurement conditions, such as ambient noise and electrode contact. Most authors did not report the actual value of the SNR, although they usually report it as poor. In experimental conditions, it is also used as the main parameter to determine the best location of the measuring electrodes (14, 55, 83, 90–93).

### 5.3 Measurement procedures

Measurement procedure describes the selection of subjects, the actual procedure of measurement, and the type of experimental meal ingested.

#### 5.3.1 Subject selection

Measured subjects are selected in compliance with specific research; however, to perform statistical comparison, the control group of healthy (26, 94) and pathological group (25, 27, 95) of subjects is needed. Many authors were interested in measurement of electrical activity of the stomach in animals, especially in dogs (96–99), pigs (81, 84, 91, 100–104), and rabbits (105, 106). Measurement with human subjects was usually aimed to pathological cases of adults (107–109) or children (66, 75, 110, 111). Most observed pathology in reviewed articles was gastroparesis and pathologies that required partial or total gastrectomy, e.g., stomach cancer (18, 25, 27, 53, 95).

#### 5.3.2 Procedure

The procedure of measurement consisted of several steps which can include ingestion of specific meal. The electrical activity of stomach is usually measured in the fasting condition (preprandial activity) first and then in the fed condition (postprandial activity). Ingested meal is used as stimuli for stomach activity, and at the same time, it is an experimental intervention required to perform statistical analysis. Many authors do measurements in both conditions with previously defined circumstances and specifically defined experimental meals (65–67, 112, 113). Because of very low frequency of gastric electrical waves, the time of measurement varies from the order of tens of minutes (50, 75, 108, 114) to few hours (94, 114–116). The stomach has a delay after ingestion of experimental meal; therefore, there should be also a pause between measurement of preprandial and postprandial activity. There were also research studies aimed to full-day measurement of electrogastrogram (9, 48, 92, 117). An example measurement procedure consists of a 60-min preprandial measurement, followed by 10 min of food consumption, a 10-min response delay, and finally a 60-min postprandial measurement.

#### 5.3.3 Experimental diet

**Figure 9:**
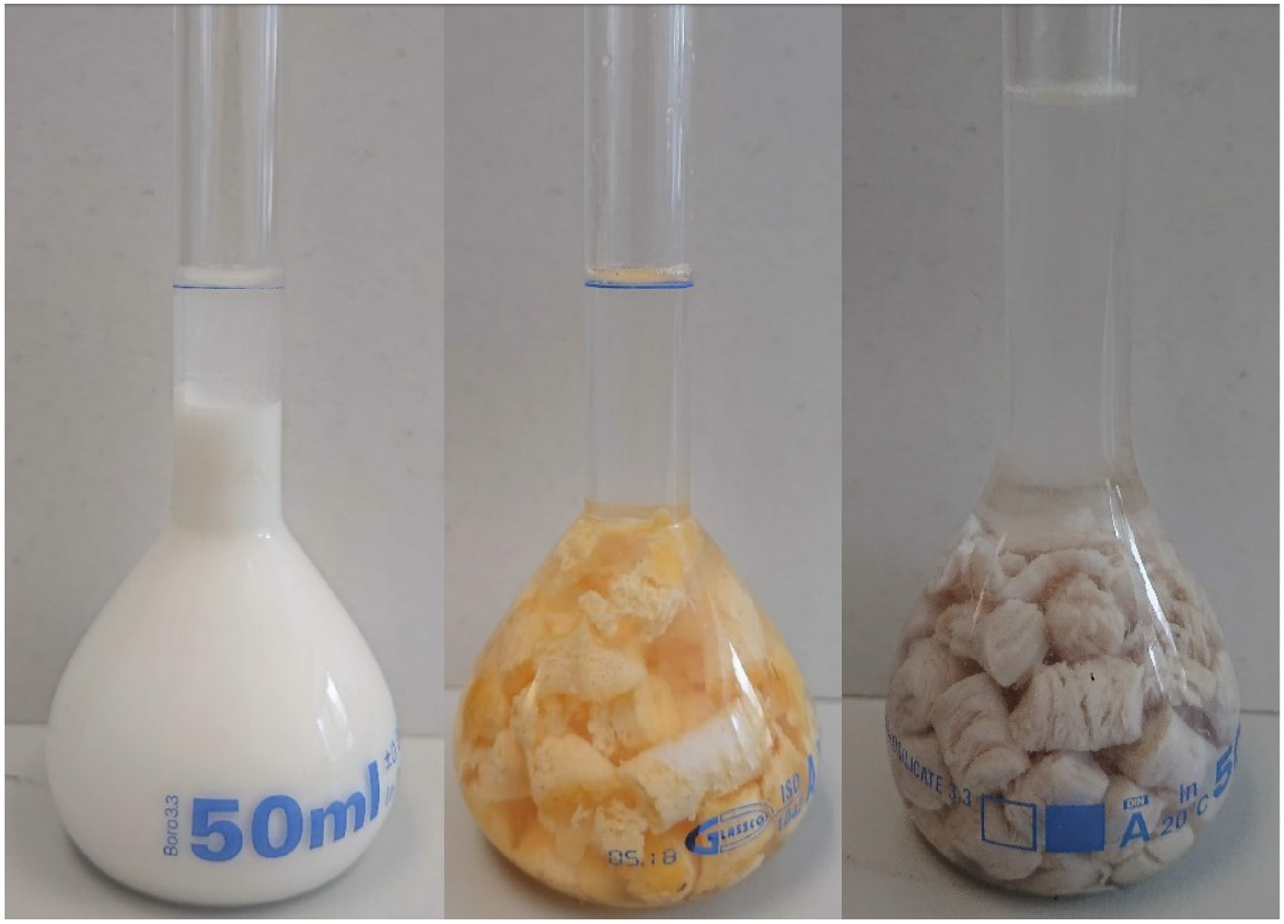
Examples of test meals measured for their density (cut into smaller pieces). From left: yogurt, scrambled eggs, chicken steak.

Experimental diet represents testing substance served to subjects as stimulus for the stomach. It could be yogurt (10, 68, 110), scrambled eggs (66, 79, 94, 118), or a type of pastry (10, 67, 119, 120). Liquids, such as water (10, 121, 122) or milk (115, 118, 123), were also examined in some research studies. However, no study defines what specific parameter of experimental diet is most significant, e.g., consistency or density. Examples of experimental diet are shown in Figure 9.

## 6 Methods of filtration and analysis

Analysis of measured signal requires filtration because signals contain noise and biological artifacts. One study was carried out to identify form of artifacts, to successfuly remove them (76). In general, the filtration is performed in frequency or time domain. Processed signals are then usually classified into bradygastria, normogastria, and tachygastria based on dominant frequency.

### 6.1 Frequency domain

Filtration in frequency domain is done using digital frequency filters which preserve only useful part of processed signal which is mainly in range between 0.5 and 9 CPM. Analysis of signal using methods based on Fourier transform usually begins with a division of signal into smaller segments which are processed resulting into chart of power spectral density which is manually evaluated by expert or automated algorithm (4, 10, 52, 68, 72, 94, 124).

### 6.2 Time domain

If used sampling rate is higher than few hertz, the high-frequency noise and very distinctive ECG interference are present in measured signal. In time domain, the filtration is usually based on convolutional methods. The noise can be removed from acquired signals by smoothing with moving average or median filter (72, 94). The smoothing algorithms are usually just initial phase of filtration process. To remove the ECG noise, the Wavelet transform is a more complex and much more efficient filtration method if wavelet is selected correctly, e.g., Morlet (34, 46) or Daubechies (58, 125, 126) wavelets.

### 6.3 Other approaches

In few cases, the machine learning algorithms, such as neural networks, were also used to process signals (11, 93, 127). Other methods such as Empirical Mode Decomposition, Hilbert-Huang transform, or Independent Component Analysis are used (119, 121, 128).

## 7 Comparative analysis of EGG procedures

Electrogastrography is a method known for long time, and many authors contributed to its development. Even if there is no firmly defined medical standard for its measurement, there is a common protocol which most authors follow. In this review, the comparison between electrode placement, leads, devices, measured procedures, and filtration and analysis methods is conducted. The aim of these comparisons is to describe and depict mostly used approaches for defined category and help future researchers to pick a suitable method.

Unlike other review articles, this study provides a comprehensive and technically oriented description of the utilized devices, the placement of electrodes, and the methods employed for signal processing. All of this information is primarily presented in Table 2, followed by subsequent figures featuring charts that illustrate the most commonly used devices, methods, and approaches. The other review articles are mostly focused on the physiological description of the origin of gastric motility, and the technical information provided by the reviewed articles is not very detailed or highly focused on only one method (132–135).

**Table 2:** Chronological listing of measurement methods, procedures, and analysis types.

**References**	**Localization**	**Apparatus**	**Electrodecount**	**Electrodetype**	**Leads**	**Samplingrate**	**Pre-processing**	**Measurementtime**	**Test diet**	**Age**	**Health**	**Analysis**
Alvarez (1)	V	G	2	Z	B	-	-	-	-	E	-	-
Brown et al. (26)	A	A	6	A	B	A	A	90/30/90	A	A	H	F
Bellahsene et al. (32)	A	A	2	A	U	~4	A	~35/-/-	C	A	H	F
Hamilton et al. (23)	A	A	2+1	A	U	-	A	~45	C	-	B	F
Geldof et al. (27)	A	A	4+2	A	M	1	A	30/4/35	C	M	B	F
Bruijs et al. (15)	A	A	4+3	A	B	1	A	240/-/90	C	-	H	F
Chen and McCallum (123)	U	A	3+1	-	U	2	A	30/-/(30,60)	C	A	H	F
Cucchiara et al. (54)	U	P	6	A	B	1	A	60/-/60	C	C	B	F
Homma et al. (56)	A	A	4	A	B	2	A	30/-/30	C	M	P	F
Atanassova et al. (96, 109)	A	C	4+1	-	B	200	T	180/30/180	C	A	H	T
Chang et al. (14)	A	C	3+1	A	U	-	F	15/5/15	C	A	H	F
Lindberg et al. (92)	A	E(1)	2+1	A	B	1	D	1440	A	A	H	F
Pfaffenbach et al. (78)	U	E(1)	2+1	A	B	4	A	60/10/60	C	A	B	F
Lin et al. (79)	A	E(1)	2+1	A	B	1	A	30/-/30	C	A	B	F
Mintchev et al. (73)	A	-	4	-	B	10	M	60	-	-	P	F
Lorenzo et al. (75)	X	-	3	-	B	10	A	30/-/30	-	C	P	F
Bonapace et al. (124)	A	E(1)	2+1	-	B	4	A	60/-/120	C	M	B	F
Levanon et al. (129)	U	E(1)	2+1	-	B	-	A	60/-/120	C	A	H	F
Parkman et al. (114)	A	E(1)	2+1	-	B	4	A	30/-/30+180	C	A	H	F
Chen et al. (110)	A	E(1)	2+1	A	B	1	A	60/-/60	C	C	B	F
Usami et al. (80)	A	P	2	-	B	-	A	70	-	A	H	F
Kaiho et al. (48)	A	E(2)	4+1	A	U	1	A	1440	A	A	B	F
Dibaise et al. (116)	A	E(1)	2+1	A	B	4	A	30/-/120	C	A	H	F
Full-Young et al. (89)	A	C	3+1	A	U	1	A	30/5/30	C	A	B	F
Mathur et al. (113)	A	A	2+1	A	B	2	A	60/-/120	C	A	P	F
Patterson et al. (52)	U	E(1)	2+1	-	B	4	A	-/30/~219	-	C	H	F
Gonlachnvit et al. (67)	A	E(1)	2+1	-	B	4	A	30/-/240	C	A	H	F
McNearney et al. (88)	A	E(1)	4+2	A	U	4	A	60/-/60	C	A	B	F
Franzese et al. (53)	U	E(1)	2+1	A	B	4	A	60/-/60	C	C	P	B
Katoh et al. (130)	A	E(2)	4+1	-	U	1	A	60/-/120	-	A	H	F
Jones et al. (65)	A	A	2+1	A	-	4	A	15/5/30	-	A	H	F
Tokumaru et al. (72)	A	A	4+1	A	B	32	T	-/120/8,16,16	C	A	H	B
Riezzo et al. (115)	U	E(1)	2+1	A	B	1	A	60/-/120	-	C	H	F
Ohtaki et al. (55)	U	P	-	-	-	2	D	30/-/30	C	C	B	B
Simonian et al. (94, 95)	A	E(1)	4+2	A	U	1	A	60/-/60	C	A	B	F
Chien-Lin et al. (62)	A	E(3)	2+1	A	B	-	A	15/5/30	C	A	P	F
Kirjavainen et al. (117)	A	P	2	-	-	100	-	1440	-	C	B	-
Filho et al. (68)	A	P	2+1	A	U	4	A	60/-/60	C	A	B	F
Borrelli et al. (111)	U	E(1)	2+1	A	B	4	A	60/-/60	C	C	P	B
Friesen et al. (66)	A	E(1)	2+1	A	-	4	A	30/10/60	C	M	H	F
Nakao et al. (77)	A	E(4)	2+1	A	B	~2	A	10	-	A	H	F
Dirgenali et al. (46)	A	E(4)	2+1	-	B	200	A	-	-	M	B	B
Fraško et al. (47)	A	E(1)	2+1	A	B	4	A	30	-	A	B	F
Gopu et al. (13)	A	C	2+1	A	B	4	A	60/-/60	C	A	P	T
Krusiec-Swidergol et al. (18)	A	E(1)	4+2	A	U	1	A	30/-/120	C	A	B	F
Sikkandar et al. (125)	A	E(4)	2+1	A	B	100	M	30/-/30	C	A	H	F
Contreras et al. (16)	A	C	6+1	-	B	10	A	15/-/30	C	A	H	F
Komorowski et al. (87)	A	C	4+2	-	U	500	D	40/15/150	C	-	-	F
Seligman et al. (7)	A	E(2)	4+1	-	U	1	-	15/-/45	C	A	P	F
Gharibans et al. (9)	X	C	25	-	-	-	M	1440	A	C	-	F
Al Kafee et al. (126)	A	E(3)	2+1	-	B	-	M	30/-/60	C	A	B	T
Waluga et al. (5)	A	P	4+2	A	U	1	M	30/30/30	C	A	H	B
Popović et al. (4)	A	A	3+2	A	-	2	A	-	-	A	H	B
Vargas-Luna et al. (118)	A	E(4)	2+1	-	B	250	M	30/-/60	C	C	B	B
Wang et al. (131)	A	E(3)	2+1	A	B	1	M	15/5/30	C	A	P	B
Calder et al. (76)	A	E(5)	64+2	A	U	-	D	10	-	A	P	B

Legend for symbols used in this table are shown in Table 3.

**Table 3:** Legend of symbols used in Table 2.

Localization V — Visual A — Anatomical approximation U — Ultrasonography X — X-ray Apparatus (Company) A — Amplifier (Bioamplifier) G — Galvanometer P — Polygraph C — Custom system E — Electrogastrograph 1 — Synectics-Medtronic, worldwide 2 — Nipro, Japan 3 — 3CPM, USA 4 — Biopac, worldwide 5 — Alimetry, New Zealand Electrode count Active + Reference	Electrode type A — Ag/AgCl Z — Zn/ZnCl Leads U — Unipolar B — Bipolar M — Mixed Sampling rate [Hz] A — Analog (infinity, e.g. magnetic tape) Pre-processing A — Analog frequency filter D — Digital frequency filter T — Time domain filter (average) M — Mixed Measurement time [min.] Preprandial/Delay(or eating)/Postprandial	Test diet C — Controlled A — Any Age C — Children A — Adult E — Elderly M — Mixed Health H — Healthy P — Pathological B — Both Analysis F — Frequency domain T — Time domain B — Both

### 7.1 Electrode placement

**Figure 10:**
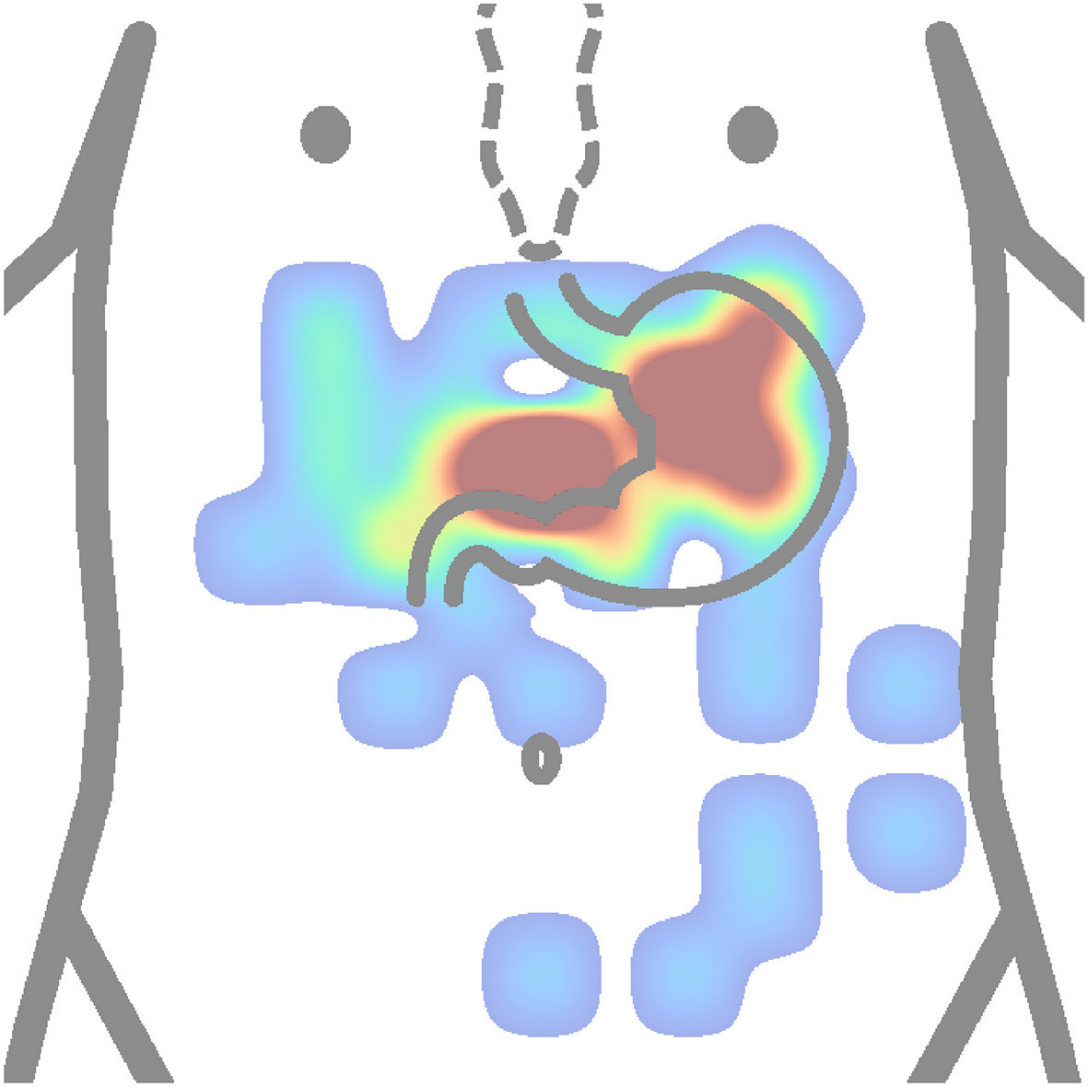
Heatmap (concentration map) of active electrode positions of all authors is presented in Table 2. The matrix layouts were excluded. Positions of active electrodes from layout of each author in the table were added to the same equidistant matrix and summed to produce this heatmap. Red color is high concentration, and blue color is low concentration.

**Figure 11:**
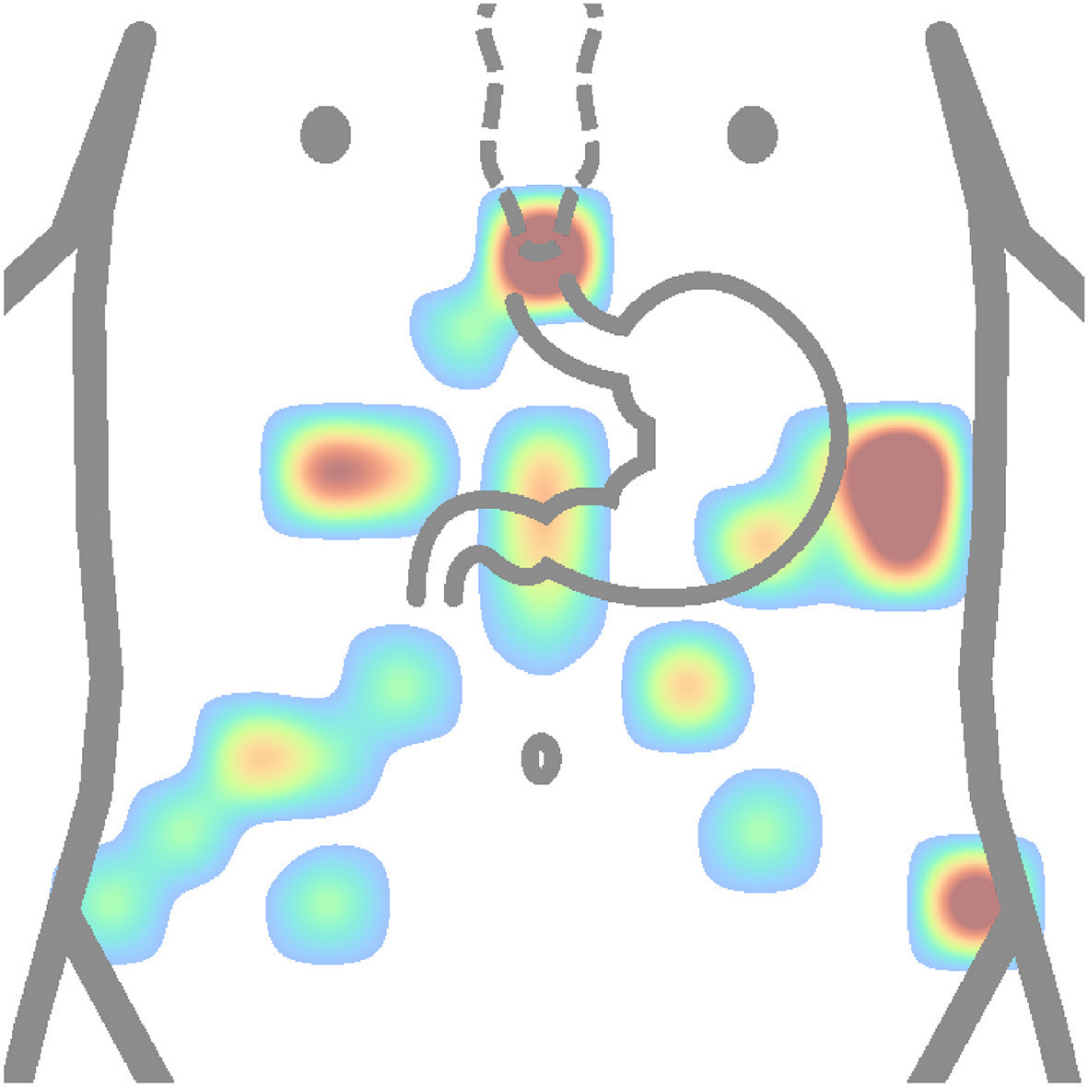
Heatmap (concentration map) of reference electrode positions of all authors is shown in Table 2. The matrix layouts were excluded. Positions of reference electrodes from layout of each author in the table were added to the same equidistant matrix and summed to produce this heatmap. Red color is high concentration, and blue color is low concentration.

First, it is necessary to find the placement of the stomach and ensure quality signal the places should be shaved, abraded, and cleaned. Then, the electrodes are placed over in a specific layout. Based on all available descriptions of actual electrode placements from reviewed authors as shown in Table 2, two heat maps were constructed. Figure 10 depicts the most used placement of active electrodes in the abdomen, and Figure 11 shows the most common locations of the reference or ground electrodes.

The active electrodes placement heat map (Figure 10) shows that authors used to place electrodes mostly to proximal (cardia) and distal (pylorus) ends of the stomach. Based on anatomical approximation, these two locations can be described as the middle between the process of sternum and the navel, and the intersection of the line constructed from the first electrode location at an approximate angle of 45 ° and perpendicularly lowered from the left nipple. Other electrode placements were more or less experimental or random. It can be observed that several electrodes are placed completely outside the stomach area.

In contrast, based on Figure 11, the most used places for reference or ground electrodes are located at the process of sternum, iliac crest, and intersection of horizontal line constructed from middle point between process of sternum and the navel and left costal margin. Of the reviewed studies by all authors shown in Table 2, the most used method of electrode placement was anatomical approximation, as 79 % of the reviewed authors chose this method for their research.

### 7.2 Leads

Usually, only a few channels are used, but even one is enough which means that the minimum number of active electrodes is 2 for the bipolar connection method. The most used number of active electrodes is 2, which is 55 % of reviewed authors, followed by the number of 4, which was used by 23 % of reviewed authors. Nevertheless, this is mostly defined by used bioamplifier and its manufacturer. The advantage of using lower number of leads is that there are less signals to process and also measurement device can be simpler. The downside is that information may be missing that we do not currently know. Leads are usually connected in bipolar way, which was used by 60 % of reviewed authors. It is more than twice as much than unipolar way, which was used by 27 % of authors from Table 2. However, this is also very affected by selection of device, aim of study, or final analysis requirements, e.g., slow wave analysis is usually done with bipolar connection while spatial mapping requires unipolar one.

### 7.3 Devices

In reviewed studies, there were several types of devices used. The majority of authors, 52 %, used commercial electrogastrographs from several companies. Mostly used devices were electrogastrographs from company Synectics, one system with one bipolar channel and second with four unipolar channels. This was 62 % from all electrogastrographs used by reviewed authors, as shown in Table 2. On the other hand, these devices are relatively old and still only experimental. Sampling rate of acquirement is usually 1 Hz or 4 Hz. Both sampling rates took 27 % from all used frequencies. However, not all authors specified the sampling rate they used or the manufacturer does not state this information publicly, and this was for 16 % of reviewed studies shown in Table 2.

### 7.4 Measurement procedures

When electrode placement and measuring apparatus are prepared, the measurement procedure must be defined. If the 4 wholeday studies are omitted, the 81 % of reviewed studies in Table 2 were consisted of preprandial measurement, serving of test meal, and postprandial measurement. There were 6 studies based only on preprandial measurement and 2 based only on postprandial measurement. Authors whose studies were consisted of preprandial, waiting and postprandial phase spent in average 51 minutes for preprandial measurement, 7 minutes for waiting phase, and 72 minutes for postprandial measurement. The majority of authors had rules that during measurement, the subject should be in supine position and should not move or speak. The majority of the authors have used controlled diet for their measurement, and it was 68 %. On the other hand, 25 % of reviewed authors did not state what diet was used or measured only preprandially.

### 7.5 Subject selection

The healthy subjects were selected in most studies, as shown in Table 2. In total, 41 % of healthy subjects, 20 % of subjects with pathology, and 34 % subjects were mixed. The rest of the authors did not specify if subjects were healthy or not. The most measured subjects were adults, 63 %, followed by children with 20 %. The 9 % of subjects were mixed and 7 % were not specified.

### 7.6 Filtration and analysis

Signals are usually pre-processed by frequency or convolutional filters, divided into segments, and evaluated based on value of dominant frequency. Even if the medical standard is not yet defined officially, it is obvious that the procedure of reliable acquirement of electrical activity of the stomach was formed during past years anyway. Below in this chapter, the overview of all authors and their research in relation to electrogastrography are introduced. Some authors or their research studies were not included into table because they have used same system or approach already included. For each author, these parameters are listed: the method of stomach localization, type of measurement apparatus, count of electrodes, electrode type, lead connection type, sampling rate, method of signal pre-processing, time of measurement, test diet, age and health of subject, and method of analysis. In total, the 56 authors interested in non-invasive electrogastrography from 1922 to 2022 are shown in Table 2.

## 8 Conclusion

Over time, there were many studies aimed to electrogastrography, and in this review, the 129 articles specifically aimed to measurement systems, procedures, and analyses were selected out of total 1,322 papers from initial search. Number of electrodes is usually as low as possible, mostly approximately 3, and measurement systems used in research and clinical practice gradually changed from general amplifier to devices, specifically designed to measure exclusively electrogastrogram. Signal pre-processing is mostly conducted by analog filter which is already at the acquisition level, and after digitalization, the signals are in most cases evaluated using spectral analysis methods. However, this approach may lead to the loss of as-yet-unknown signal components, while from a research perspective, universal bioamplifiers still offer wider possibilities for signal analysis. In clinical practice, however, complete electrogastrographs are of course more suitable. In most cases, experimental studies aimed to compare between pathological and control groups. The majority of studies were interested in examination of subjects from age group of adults or children. The test meal is usually carefully controlled and prepared, but most studies did not wait for the gastric response to the ingested meal and measured immediately, which could affect the quality of postprandial signals. Studies with a high sampling rate required a more complex filtering process than studies with a lower one due to the presence of higher frequency noise. On the other hand, studies with a lower sampling frequency did not need such complex pre-processing, but the information hidden in the higher frequency component could be lost. Currently, measurement devices are readily available, and processing methods are sufficiently efficient to obtain a quality signal that can be analyzed for the purpose of diagnosing specific pathologies in the human stomach. With the current possibilities and trends, there will likely be an expansion of wearable devices capable of measuring EGG over long-time period. With such a significant amount of acquired data, there will be an opportunity to process the data easily using artificial intelligence methods without the necessity of fully understanding the signal.

## Data availability statement

The raw data supporting the conclusions of this article will be made available by the authors, without undue reservation.

## Author contributions

DO: Formal analysis, Methodology, Software, Writing – original draft. MA: Data curation, Resources, Supervision, Writing – original draft. MP: Formal analysis, Funding acquisition, Resources, Visualization, Writing – review & editing. JK: Conceptualization, Resources, Validation, Writing – original draft.
